# Synthesis and properties of porous CLEAs lipase by the calcium carbonate template method and its application in biodiesel production

**DOI:** 10.1039/c9ra04365a

**Published:** 2019-09-19

**Authors:** Changlin Miao, Huiwen Li, Xinshu Zhuang, Zhongming Wang, Lingmei Yang, Pengmei Lv, Wen Luo

**Affiliations:** Guangzhou Institute of Energy Conversion, Chinese Academy of Sciences Guangzhou 510640 China yanglm@ms.giec.ac.cn lvpm@ms.giec.ac.cn +86 20 87065195 +86 20 87057727 +86 20 87057760; Key Laboratory of Renewable Energy, Chinese Academy of Sciences Guangzhou 510640 China; Guangdong Key Laboratory of New and Renewable Energy Research and Development Guangzhou 510640 China; Collaborative Innovation Center of Biomass Energy Zhengzhou 450002 Henan Province China

## Abstract

In this work, porous cross-linked enzyme aggregates (p-CLEAs) were synthesized by the *in situ* co-precipitation method using CaCO_3_ microparticles as templates. The preparation procedure involved the immobilization of crude lipase as CLEAs *via* precipitation with ammonium sulfate and entrapping these lipase molecules into the CaCO_3_ templates, followed by DTT (dithiothreitol)-induced assembly of lipase molecules to form lipase microparticles (lipase molecules were assembled into microparticles internally using disulfide bonds within the lipase molecules as the molecular linkers and stimulated by dithiothreitol); finally, the removal of CaCO_3_ templates was performed by EDTA to form pores in CLEAs. The scanning electron microscopy analysis of p-CLEAs showed a porous structure. p-CLEAs showed obvious improvement in thermal stability (after incubation at 65 °C, p-CLEAs lipase retained 86% relative activity, while free lipase retained only 33.67%) and pH stability (p-CLEAs relative activity was over 90% while for free lipase, the relative activity ranged from 72% to 89% from pH 6 to 9) than free lipase and could hold relatively high activity retention without activity loss at 4 °C for more than six months. The application of p-CLEAs in producing biodiesel showed a higher degree of conversion. The conversion of fatty acid methyl ester (FAME) was 89.7%; this value was higher by approximately 7.4% compared to that of the conventional CLEAs under the optimized conditions of a methanol–oil molar ratio of 6 : 1, with a p-CLEAs lipase dose of 20% and water content of 3% at 45 °C for 24 h. The FAME conversion remained greater than 70% even after reusing the p-CLEAs lipase for 8 reactions. The results demonstrated that the p-CLEAs lipase is suitable for applications in the preparation of biodiesel.

## Abstract 2

Porous cross-linked enzyme aggregates (p-CLEAs) were synthesized. This p-CLEAs presented a complete structure with abundant channels, large specific surface and more efficient catalytic effect compared with conventional CLEAs.

## Graphical abstract

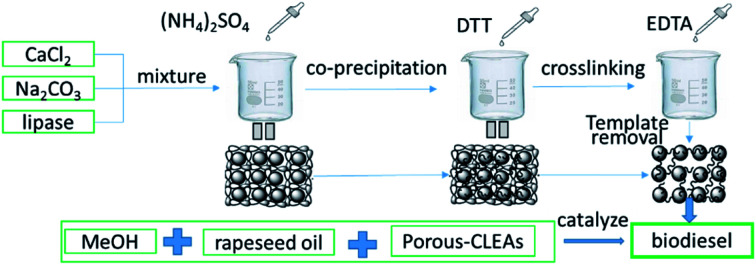

## 1. Introduction

Lipase catalysis has attracted increasing attention due to its mild reaction conditions, strong specificity and high efficiency.^1^ However, free lipase is usually susceptible to temperature or pH changes and has poor reusability.^2^ Therefore, lipase immobilization has been frequently used to overcome these shortcomings and to provide easily reusable biocatalysts.^3^ The immobilization of lipase gives the enzyme greater operational stability and durability and in some cases makes it possible to use it in continuous processes, thus enabling the use of biocatalysts at an industrial scale.^4^ Immobilization usually involves binding lipases to a carrier (support),^5^ but^6^ a serious drawback of such carrier-bound lipases in general is their low catalytic activity owing to the presence of a large proportion of non-catalytic carriers (often >95% of the total mass, which causes the dilution of lipase volumetric activity).^7^ Furthermore, enzymes have a certain superior space structure, which is influenced easily by the carrier's surface physical properties and chemical grafting. The influence of such factors causes irreversible damage to the spatial structure or the active center of the enzymes, which results in the reduction of enzyme activity or inactivation, thus seriously affecting the effects and the use of immobilized enzymes.^8^ In contrast, immobilization by cross-linked enzyme aggregates (CLEAs) molecules provides high productivity without carrier-immobilized enzymes and avoids the influence of the carriers.^9^ The cross-linked enzyme aggregates (CLEAs)^10^ technique is a method for the preparation of immobilized enzymes by the use of a crosslinking agent for the formation of enzyme aggregates, which does not need other carriers; therefore, the catalytic activity per unit volume is very high, which maximizes the volumetric catalytic activity and space-time productivity.^11^

However, even though CLEAs show promise, there are some limitations:^12^ some common problems with CLEAs are their irregular shapes, uncertain physical forms, wide range of particle size distribution, compact surfaces, small surface areas, and small pore structures.^13^ The process of CLEAs catalysis which may lead to slow diffusion of substrates restriction, space resistance and distribution. Therefore, these shortcomings may affect the catalytic properties of enzymes, thus their efficiency.^14^

Generally, size, porosity, and surface area are necessary characteristics to ensure the bioactivity and efficiency of lipase particles.^15^ The pore size and pore size distributions of the immobilized lipase have a strong effect on the easy transport of bulky molecules into its active site and importantly, this creates an environment most favorable for the expression of lipase activity.^16^ The porous structure of CLEAs lipase is effective to reduce or eliminate the undesired diffusion restriction, which can provide active sites for the molecules that are too large to enter the pores of porous CLEAs (p-CLEAs), decreasing the diffusional resistance for the reaction of incoming and outgoing species at the active sites.^17^ Therefore, the reactant molecules can easily diffuse to the active sites, which leads to higher catalyst activity.^18^

Nevertheless, studies on the control of lipase particle size and porosity in the CLEAs process and the effect of particle size and porosity on the lipase activity have rarely been reported.^19^ In this paper, CLEAs with a controlled particle size and pore structure were synthesized *via* combined CaCO_3_ templating and co-precipitation processing.^20,21^ Then, the synthesized p-CLEAs lipase was characterized using FT-IR and SEM techniques. Finally, the application of the p-CLEAs lipase in biodiesel synthesis was discussed. The effects of various reaction parameters, including reaction time and amount of the p-CLEAs catalyst, on the biodiesel conversion rate were investigated in this experiment.

## 2. Experimental details

### 2.1 Materials and reagents

#### 2.1.1 Materials

Glutaraldehyde, dithiothreitol (DTT), calcium chloride, sodium carbonate, and ethylene diamine tetraacetic acid (EDTA) were purchased from Sigma-Aldrich. Free lipase (CALB) from the transgenic *Candida antarctica* was donated by Novozymes of Denmark. Methanol was purchased from Tianjin Damao Chemical Co. Rapeseed oil was purchased from the local market. All other chemicals and reagents used were of analytical grade and used as such without further purification.

### 2.2 Preparation of conventional CLEAs lipase

The CLEAs lipase was prepared according to published ref. 22 and 23 by the conventional method consisting of precipitation, cross-linking, and washing. Briefly, in a 50 mL centrifuge tube with a magnetic stirrer bar, 5 mL of free lipase (10 mg mL^−1^) in 0.1 M phosphate buffer (pH 7.0) solution was slowly added to 5 mL of ammonium sulfate. Then, this mixture was kept under constant agitation at 300 rpm for 45 min at 4 °C. At the same time, the sample was withdrawn from the test tube before and after precipitation, and the lipase activity was assayed. The precipitates thus obtained were then chemically crosslinked using a certain amount of glutaraldehyde (25% v/v), which was added dropwise to achieve the final concentration of 2%. The mixture was agitated in an ice bath at 4 °C for 3 h. A sample was withdrawn from the suspension containing CLEAs as well as the residual free lipase, and the lipase activity was assayed. Finally, the CLEAs were recovered by centrifugation at 4000 rpm for 10 min. The supernatant was then discarded and the precipitated CLEAs were washed with 300 mL distilled water to ensure the removal of impurities and unbound lipase. Finally, the CLEAs were dried and then stored in a closed flask at 4 °C.

### 2.3 Preparation of p-CLEAs lipase

The fabrication process steps of porous CLEAs lipase include entrapping the free lipase molecules into CaCO_3_ microparticles (templates), DTT-induced assembly of free lipase molecules to form crosslinking self-assembled immobilized lipase microparticles, and finally CaCO_3_ template removal.

#### 2.3.1 Preparation of template lipase co-precipitation granules

In a 50 mL centrifuge tube with a magnetic stirrer bar, a 5 mL lipase solution with a concentration of 10 mg mL^−1^ was formed in the phosphate buffer solution (0.1 M, pH 7.0). Then, we dropwise added the same amount, *i.e.*, 10 mL 0.3 mol L^−1^ calcium chloride and sodium carbonate. At the same time, 5 mL of ammonium sulfate was added dropwise and kept under constant agitation at 300 rpm for 5 min at 4 °C to produce the CaCO_3_ template lipase co-precipitation granules. After this, the stirring was stopped and the reaction mixture was held stationary for 2 h; during this stationary process, the formed amorphous primary precipitate of CaCO_3_ transformed slowly into spherical microparticles. Meanwhile, through co-precipitation, the free lipase molecules uniformly dispersed into the pores of the CaCO_3_ particles to form lipase-loaded CaCO_3_ spherical microparticles. Finally, the lipase-loaded CaCO_3_ spherical microparticles were washed using phosphate buffer solution five times by centrifugation (500 rpm, 2 min) and re-dispersion cycles^24,25^ to ensure the removal of impurities.

#### 2.3.2 The covalent crosslinking of lipase template co-precipitation molecules

The resulting lipase-loaded CaCO_3_ microparticles were incubated with slow shocks to open the disulfide bond of the lipase molecule with 0.05 mM DTT solutions at pH 7.5 and 4 °C for 1–2 min and were then kept stationary for 5–10 min. Subsequently, the lipase-loaded CaCO_3_ microparticles were washed using phosphate buffer solution (pH 7.5) five times to remove the DTT and impurities, re-suspended in a buffer solution (pH 7.5), and incubated for 1 h. In this way, the precipitation of intermolecular crosslinked self-assembled immobilized lipase *via* the disulfide bond between the polymerized lipase molecules occurred.

#### 2.3.3 CaCO_3_ template removal

EDTA (0.25 mol L^−1^) was added into the intermolecular crosslinked self-assembled immobilized lipase precipitated microparticles and incubated at room temperature for 1 h to remove the CaCO_3_ templates. The resulting lipase microparticles were washed using phosphate buffer solution five times by centrifugation (500 rpm, 2 min) and re-dispersion cycles. The porous immobilized lipase microspheres were obtained, which were marked as p-CLEAs.

**Fig. 1:**
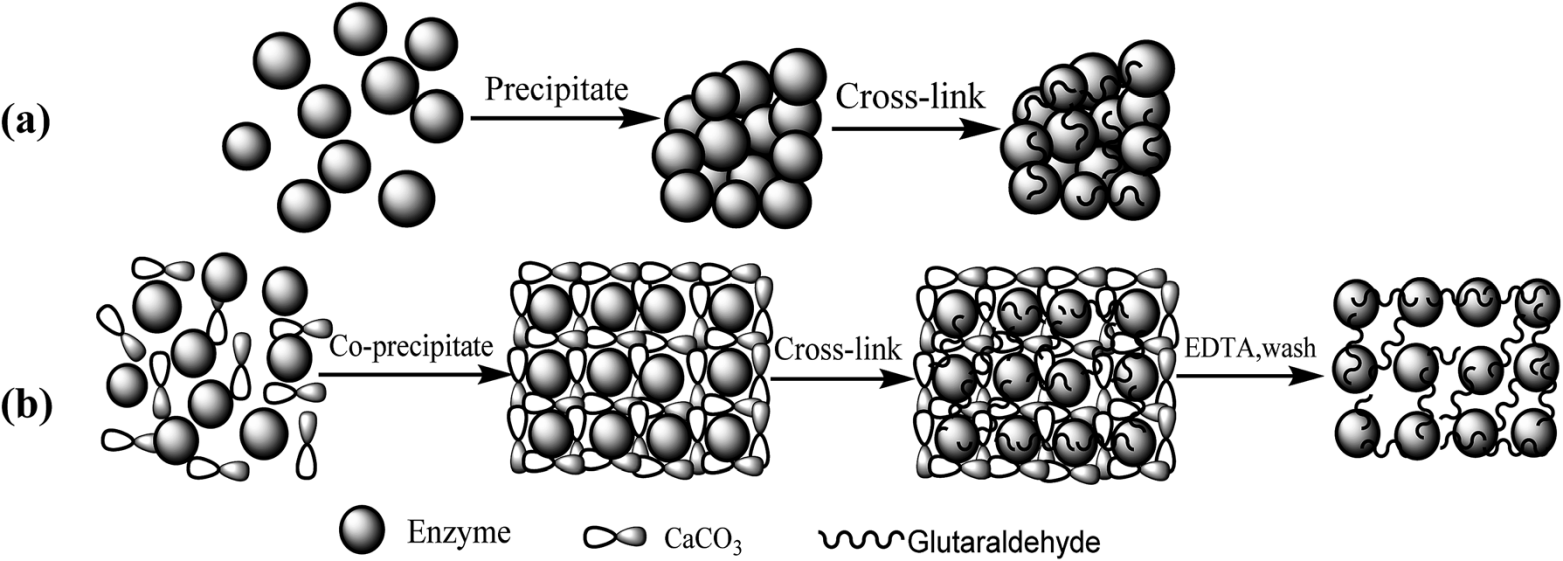
Schematic diagram of (a) CLEAs and (b) p-CLEAs preparation.

The routes for (a) conventional CLEAs and (b) p-CLEAs preparation are shown in Fig. 1.

### 2.4 Lipase activity assay

The enzymatic activities of CLEAs, p-CLEAs, and free lipase were assayed using rapeseed oil emulsion containing 3% (w/v) polyvinyl alcohol (PVA) as the substrate. A certain amount of free lipase, CLEAs, or p-CLEAs was added to 4 mL of the emulsion and 5 mL of the phosphate buffer (0.025 M, pH 7.0). The hydrolysis reaction was carried out at 45 °C for 15 min. The quantity of the fatty acid liberated was measured by titration with 0.1 M KOH solution. One international unit of lipase activity (IU) was defined as the amount of lipase producing 1 μmol of oleic acid per minute under the assay conditions.^26^

### 2.5 Characterization of CLEAs and p-CLEAs

The surface morphologies of CLEAs and p-CLEAs were examined by scanning electron microscopy (SEM) using Hitachi Japan S-3400N II SEM operated at 5 kV. The CLEAs and p-CLEAs samples were obtained by rinsing with anhydrous acetone, placed on a sample holder, and coated with platinum before being scanned under vacuum. Fourier-transform infrared (FT-IR) spectra of CLEAs and p-CLEAs were obtained on a Thermo Finnegan Nicolet 6700 FT-IR infrared spectrometer from 4000 to 400 cm^−1^ using the KBr pellet technique. The diffusion of macromolecules into the porous CLEAs lipase was studied by confocal laser scanning microscopy (CLSM) using Zeiss LSM700 CLSM (Germany) with a TCLS system attached to an inverted microscope equipped with a 40× or 100× oil immersion objective.

### 2.6 CLEAs and p-CLEAs catalysis transesterification reaction

Rapeseed oil (10 g) and different molar ratios of methanol were added into a 50 mL screw-capped vial, followed by the addition of different amounts of CLEAs or p-CLEAs. The mixtures were incubated at 45 °C with constant shaking at 250 rpm in an orbital shaking water bath at different reaction temperatures and reaction times. Once the transesterification reaction was complete, the immobilized lipase and glycerol were separated by centrifugation from the reaction mixture, and the top layer was distilled under vacuum to eliminate excess methanol.^3^ A sample was diluted with hexane and then mixed with methyl heptadecanoate as an internal standard; the conversion was analyzed by gas chromatography (GC). The reactions were carried out in duplicate, and the conversion between the duplicates was found to agree within 2%.

The fatty acid methyl esters in biodiesel were analyzed using a Shimadzu Gas Chromatograph (GC-2010) equipped with an AOC-20i automatic injection port with a flame ionization detector (FID). The capillary column was DB-WAX (length 30 m, internal diameter 0.25 mm). Test conditions: He carrier gas, flow rate 1.0 mL min^−1^, H_2_ flow rate 40 mL min^−1^; air velocity 30 mL min^−1^; injector temperature 280 °C; detector temperature 300 °C; programmed temperature, oven initiating temperature 80 °C, which was elevated to 250 °C at a rate of 12 °C min^−1^, increased up to 300 °C at 32 °C min^−1^, and then kept constant for two minutes; split sampling, with the split ratio of 1 : 20, sample size 1 μL.^27^ The biodiesel conversion was calculated in terms of the weight of the alkyl esters obtained per unit weight of the oil. The resulting methyl ester peaks in the sample were identified by comparing their retention time with standards of known composition.

## 3. Results and discussion

### 3.1 Properties of p-CLEAs lipase

**Fig. 2:**
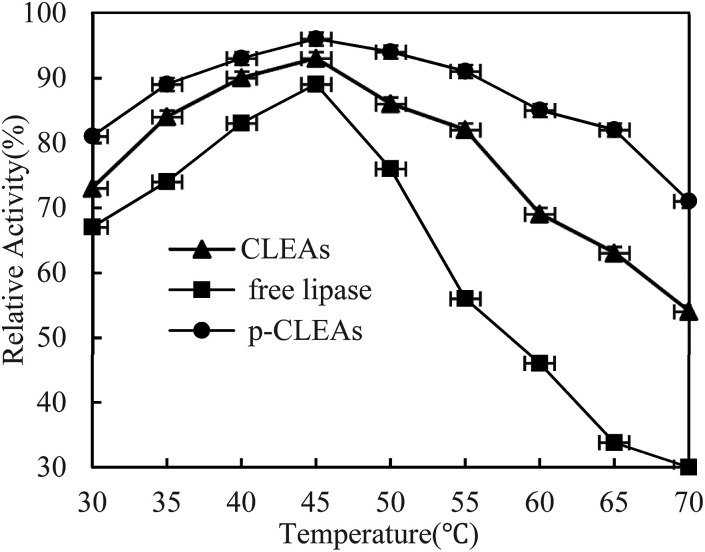
Effect of temperature on the activity of free lipase, conventional CLEAs, and p-CLEAs.

The thermal stability of free lipase, conventional CLEAs lipase, and p-CLEAs lipase was determined by incubating the lipase samples in phosphate buffer (0.1 M, pH 7.0) without the substrate from 30 °C to 70 °C in a temperature-controlled water bath for 30 min. After incubation, the activities of free lipase, conventional CLEAs, and p-CLEAs were determined immediately. The results are shown in Fig. 2.

As shown in the figure, the activity of both the free and immobilized lipases is closely related to temperature. The free and immobilized lipases displayed the highest activity at approximately 45 °C. Besides, a significant difference was observed among the relative enzyme activity values of free lipase, conventional CLEAs lipase, and p-CLEAs lipase at all the tested temperatures. Compared with the free lipases, the conventional CLEAs lipase and p-CLEAs lipase had improved thermal stability in a wide range of temperature distributions. After incubation at 65 °C, the conventional CLEAs lipase and p-CLEAs lipase retained 63% and 86% relative activities, respectively, while free lipase retained only 33.67% relative activity. These results showed that the CLEAs lipase and p-CLEAs lipase could endow the immobilized lipase with more pronounced protection from thermal denaturation. Furthermore, we further compared the conventional CLEAs lipase and p-CLEAs; p-CLEAs retained about 81% initial relative activity at 70 °C incubation, while the conventional CLEAs residual activity was about 54%, which was significantly lesser than that of the p-CLEAs residual activity. On investigating its reason, it was found that this enhanced thermal stability of p-CLEAs might be due to the formation of intermolecular disulfide bonds induced by the intramolecular disulfide bonds within the lipase molecules, thus leading to more stable covalent cross-linking of the lipase molecules among the lipase aggregates.^28^ The high stability of p-CLEAs might be useful for large-scale industrial applications under extreme conditions.

The pH dependence of the activity of free lipase, conventional CLEAs lipase, and p-CLEAs in the pH range from 5.5 to 9 was also investigated in 0.1 M phosphate buffer incubated for 5 h at 45 °C.

**Fig. 3:**
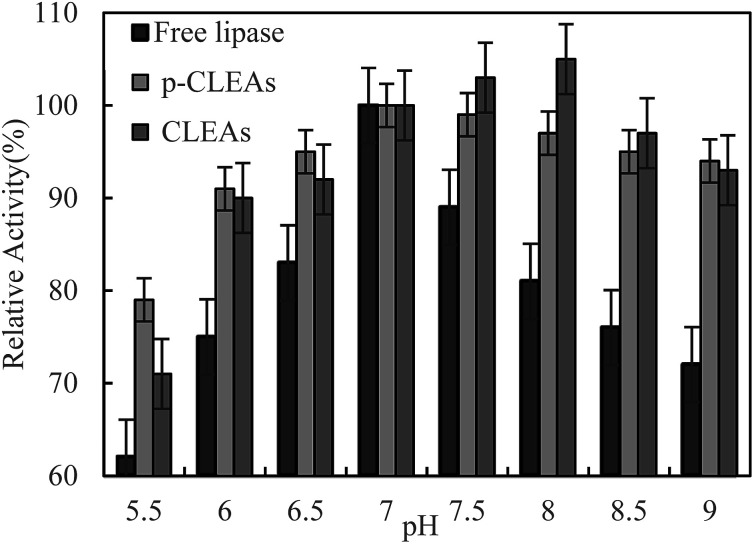
Effect of pH on the activity of free lipase, conventional CLEAs, and p-CLEAs.

As shown in Fig. 3, maximum activity is observed at pH 7.0 for both the free and p-CLEAs lipases. This indicated that the immobilization of p-CLEAs lipase had no significant effect on the optimal pH value of enzyme activity. However, due to the immobilization of lipase, higher activity was retained than that of free lipase when the pH was far from the optimal pH; the relative activities of the conventional CLEAs lipase and p-CLEAs were both over 90% while for free lipase, the relative activity ranged from 72% to 89% in the same environment with the pH values ranging from 6 to 9. The higher stabilities of the conventional CLEAs lipase and p-CLEAs than that of free lipase might be related to the more stable molecular structure induced by lipase cross-linking. In addition, the pH stability of the conventional CLEAs lipase and p-CLEAs was also evaluated. For the conventional CLEAs, the optimal pH value (pH 8.0) shifted to alkaline conditions; this might be because glutaraldehyde coupled and cross-linked all the amino acid groups on the surface of lipase, which conferred the lipase surface with a negative charge and ultimately shifted the optimum pH to higher values.^29^

**Fig. 4:**
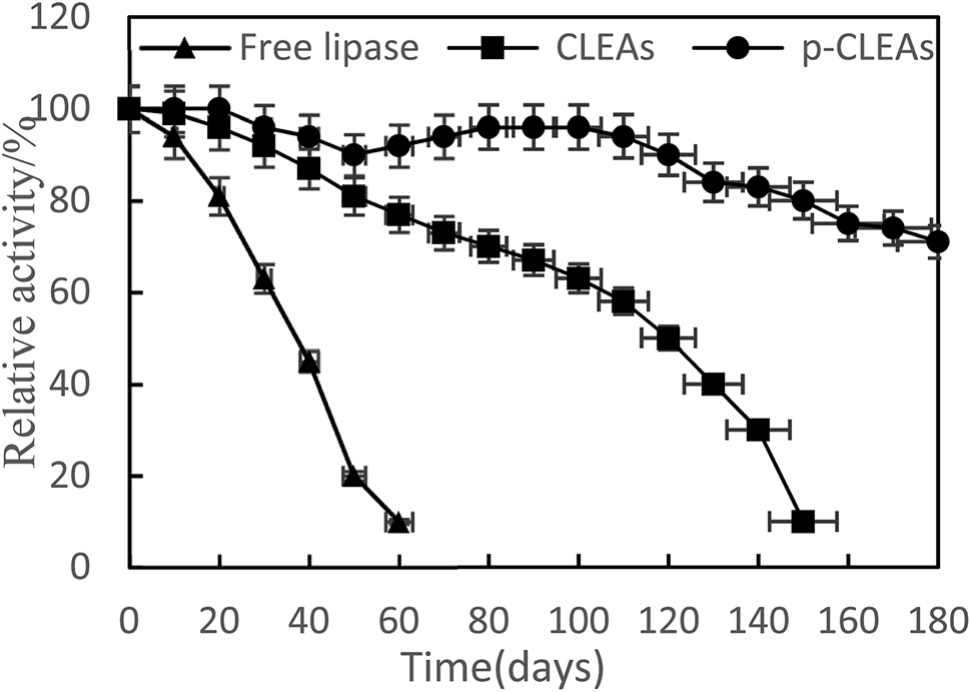
Storage stability of free lipase, conventional CLEAs, and p-CLEAs.

The storage stabilities of free lipase, conventional CLEAs, and p-CLEAs lipase were evaluated by incubating the lipase samples in phosphate buffer (pH 7) at 4 °C, and their activities were analyzed every 10 days for 180 days. The activity at 0 min was taken as 100%, and the results are shown in Fig. 4.

As indicated in this figure, the activities of the conventional CLEAs and p-CLEAs lipases show no significant decrease during the storage periods from 0 to 60 days. However, under the same storage conditions, the activity of free lipase decreased significantly and the initial activity decreased by more than 90% after 60 days. Therefore, compared with free lipase, the immobilized conventional CLEAs and p-CLEAs lipases exhibited better storage stabilities. On investigating its reason, it was found that the enhanced storage stability of the conventional CLEAs and p-CLEAs lipases might be due to the formation of covalent bonds, thus leading to more stable cross-linking, which restricts the movement of the lipases. Furthermore, we compared the conventional CLEAs and p-CLEAs lipases. The residual activity of conventional CLEAs was about 10%, while p-CLEAs retained about 71% of the initial relative activity at 4 °C incubation after 180 days of storage, which was significantly higher than the residual activity of conventional CLEAs. Moreover, after 60 days of storage, the p-CLEAs activity increased slightly with storage time and was the highest in the duration of 80–100 days. This increase in activity could be explained by the minimum possible distortion effects caused by the buffer solution on the active sites of lipases or due to the formation of stable intermolecular disulfide bonds between the protein molecules, due to which they were not readily biodegradable. Therefore, the p-CLEAs lipase had significantly higher storage stability than the CLEAs lipase, which could broaden the application of p-CLEAs lipases in industrial production.

Lipase is often poisoned by organic solvents in esterification and transesterification. In this paper, free lipase, CLEAs, and p-CLEAs were treated with methanol phosphate buffer with a concentration of 10% to study the tolerance of lipase to methanol.

**Fig. 5:**
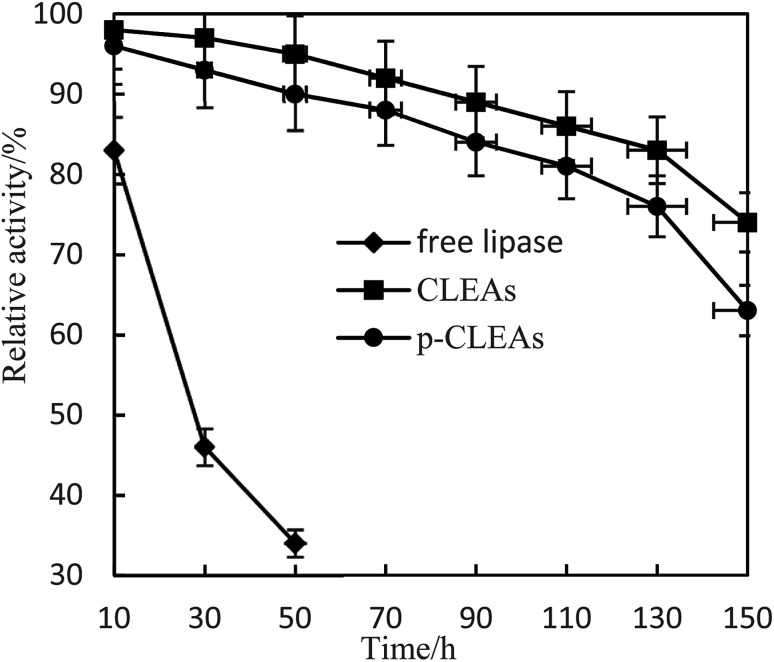
Effect of methanol on the activity of free lipase, conventional CLEAs, and p-CLEAs.

Fig. 5 shows that CLEAs and p-CLEAs have strong tolerance to methanol. After methanol treatment for 150 h, the relative enzyme activities of CLEAs and p-CLEAs were 83% and 76%, respectively, while that of the free lipase after methanol treatment for 50 h was only 34%. This may be due to the formation of a supramolecular structure after crosslinking, which weakens the effect of methanol on the active center of the enzyme and makes the enzyme activity of the crosslinked enzyme relatively higher. However, the free lipase enzyme protein was completely exposed to the environment and directly contacted with methanol; the active center of the enzyme was destroyed and thus, the enzyme activity was greatly lost. In addition, compared with the methanol tolerance of CLEAs, the methanol tolerance of p-CLEAs was also reduced to some extent; this may be because p-CLEAs have abundant pores and methyl ester has a larger interaction area with p-CLEAs, leading to faster loss of enzyme activity, but the overall decrease was small. Therefore, the tolerance of p-CLEAs to methanol extends the application of lipases in esterification and transesterification reactions.

### 3.2 SEM analysis

**Fig. 6:**
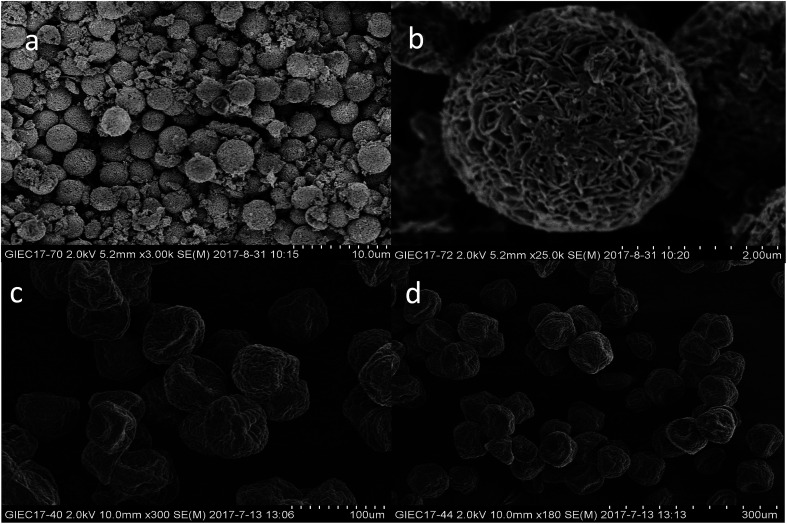
SEM analyses. (a and b) SEM images of p-CLEAs lipase; (c and d) SEM images of the conventional CLEAs lipase.

Shape, size, and morphology are important properties of CLEAs, which have important implications for industrial applications in biotransformation and can significantly affect the activity of CLEAs. In order to assess the surface morphologies of conventional CLEAs lipase and p-CLEAs lipase, they were characterized by SEM. The magnified SEM images of the p-CLEAs lipase are shown in Fig. 6a and b.

The SEM images revealed that the surface structure of the p-CLEAs lipase is different from that of the conventional CLEAs lipase. The surface of conventional CLEAs was comparatively smooth and had almost no porous spherical structures. The average size of the presented CLEAs lipase particles was 50 ± 0.95 μm and the particle size varied from 40 μm to 70 μm (Fig. 6c and d). It was speculated that this smooth, non-porous, and large particle structure of CLEAs may have a serious steric hindrance effect on the substrates and show smaller substrate conversion rates by limiting the entry of the substrate reactant to the inner CLEAs due to diffusional restrictions. On the contrary, the surface of the p-CLEAs lipase, which was prepared by CaCO_3_ as the pore-forming agent, exhibited structures having interconnected channels with pore diameters of 10–100 nm held together by a crosslinked lipase network with an average particle size of 5 ± 0.95 μm; the particle size varied from 4 μm to 6 μm. Compared with larger particles and size distributions conventional CLEAs lipase, the p-CLEAs show a narrow size distribution and smaller particles. In addition, p-CLEAs has a finer internal pore structure and higher internal surface area, making it easier for substrate molecules to enter the lipase catalysis site, which not only reduces the mass transfer restriction, but also improves the catalytic efficiency of p-CLEAs particles.^25^

### 3.3 CLSM analysis

In order to further characterize the porosity of the obtained p-CLEAs lipase, we conducted the diffusion of macromolecules into p-CLEAs lipase by CLSM with fluorescently labeled high-molecular-weight dextrans and nanoscopic fluorescent polystyrene beads. The molecular weights of the dextrans were 500 kDa and 2000 kDa with diameters of 29 nm and 56 nm, respectively. The diameter of the nanoscopic fluorescent polystyrene beads was 75–100 nm.

**Fig. 7:**
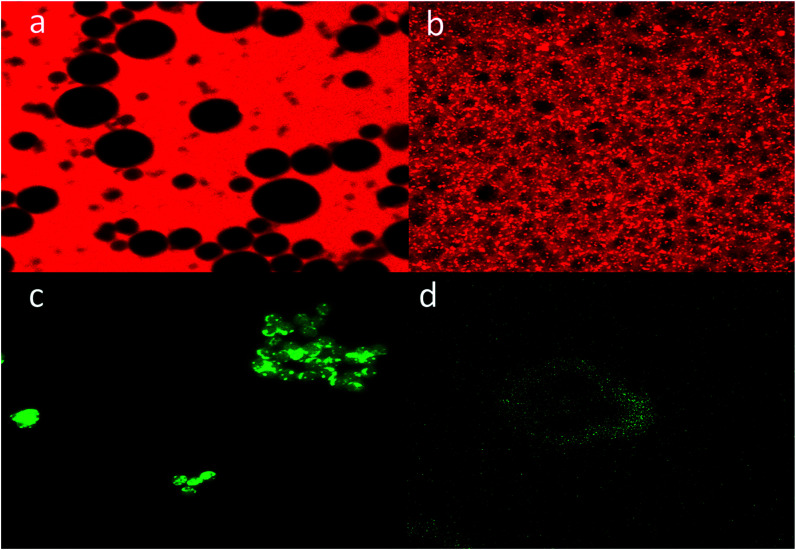
CLSM analysis. (a) The 500 kDa dextran could not diffuse into the CLEAs lipase particles. (b) The 2000 kDa dextran could freely diffuse into the p-CLEAs lipase particles. (c) The 75 nm polystyrene beads could penetrate into the interior of p-CLEAs. (d) The 100 nm polystyrene beads were stuck to the surface of p-CLEAs.

As shown in Fig. 7, the experiments reveal that even the dextran with a molecular weight below 500 kDa (29 nm) cannot diffuse freely into the CLEAs lipase particles, while the dextran with a molecular weight of 2000 kDa (56 nm) can freely diffuse into the p-CLEAs lipase particles. In addition, a permeability study with nanoscopic fluorescent polystyrene beads showed that the 75 nm polystyrene beads could penetrate into the interior of p-CLEAs, while the 100 nm polystyrene beads could not enter p-CLEAs and were stuck to the surface. This indicated that the p-CLEAs lipase particles were mesoporous with a pore diameter below 100 nm. This again confirms the above SEM analysis that verifies the porous structure of the p-CLEAs lipase. The porous structure of the p-CLEAs lipase is conducive for the diffusion of reactants and accelerates the reaction.

### 3.4 FT-IR analysis

**Fig. 8:**
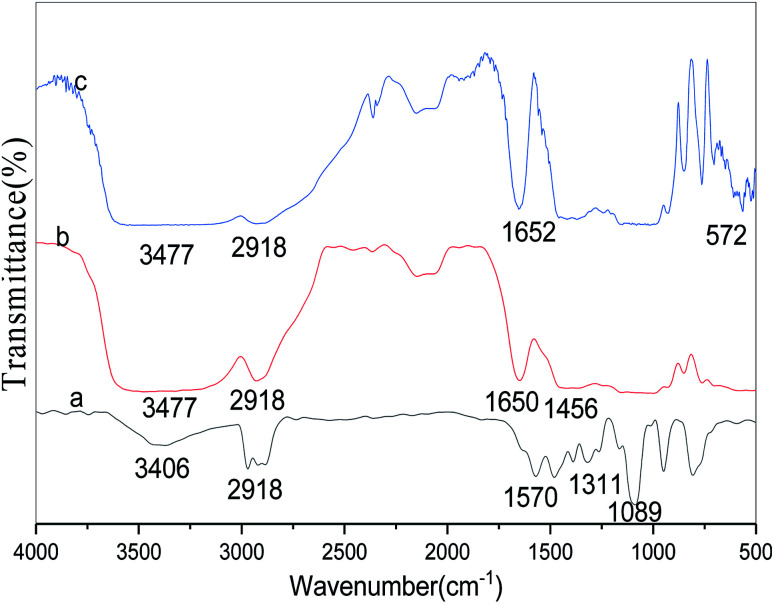
FT-IR spectra of (a) free lipase, (b) CLEAs lipase, and (c) p-CLEAs lipase.

The FT-IR spectra of free lipase, conventional CLEAs lipase, and p-CLEAs lipase are presented in Fig. 8a–c, respectively.

For the tested sample (spectrum a in Fig. 8), the absorption peak at 3406 or 3477 cm^−1^ corresponds to the hydroxyl group, and the absorption peak at 2918 cm^−1^ is ascribed to the CH_2_ bond stretching vibration for the lipase molecules; the absorption peak at 1311 cm^−1^ is due to the contribution of the bending and rocking vibrations of CH_2_. The absorption band at 572 cm^−1^, attributed to the disulfide bonds (S–S) from the p-CLEAs lipase sample, suggests that the p-CLEAs lipase has been successfully prepared.^28^ The absorption peak at 1456 cm^−1^ for the C=N vibration appeared due to the propyl chain of glutaraldehyde. These results show that glutaraldehyde, as a cross-linking reagent, has two aldehyde groups that can react with the terminal amine group of the lipase during the CLEAs process; Schiff base linkages were successfully formed between the amino groups of the lipase and the aldehyde groups of glutaraldehyde. Finally, it was confirmed that the CLEAs lipase has also been successfully prepared.

The amide band is the most sensitive spectral region of the structural components of lipase, and the structural features of lipase such as α-helix and β-sheet can be interpreted from the amide I and amide II bands in the regions of 1700–1600 cm^−1^ and 1600–1500 cm^−1^, respectively. In this study, the absorption peaks of the p-CLEAs lipase and the CLEAs lipase were similar, and no significant spectral shift for the protein amide I band and amide II band was seen. The peaks for free lipase, p-CLEAs lipase, and CLEAs lipase also showed the same typical absorption at ∼1650 cm^−1^, which is known to be assigned to amide I and corresponds to the C=O stretching vibration band. However, compared to the observations for free lipase, the absorption intensity and the area ratio of amide in the spectra of p-CLEAs lipase and CLEAs lipase were significantly higher. This result indicated that the percentage of the α-helix and β-sheet structures present in p-CLEAs lipase and CLEAs lipase increased compared to that of free lipase. Also, α-helix and β-sheet structures have many H-bonds, which can provide the secondary structure with certain stability and rigidity. Thus, after crosslinking, the secondary structure stability and rigidity of the p-CLEAs lipase and CLEAs lipase increased. This is one possible reason why the stability of the p-CLEAs lipase and CLEAs lipase and their activities were greatly improved.^8^

### 3.5 P-CLEAs lipase catalytic performance

#### 3.5.1 Effect of reaction parameters on FAME conversion

In order to investigate the performance of the p-CLEAs lipase, the effects of reaction time, catalyst dosage, methanol/oil molar ratio, and water amount on the conversion rate of biodiesel were investigated.

**Fig. 9:**
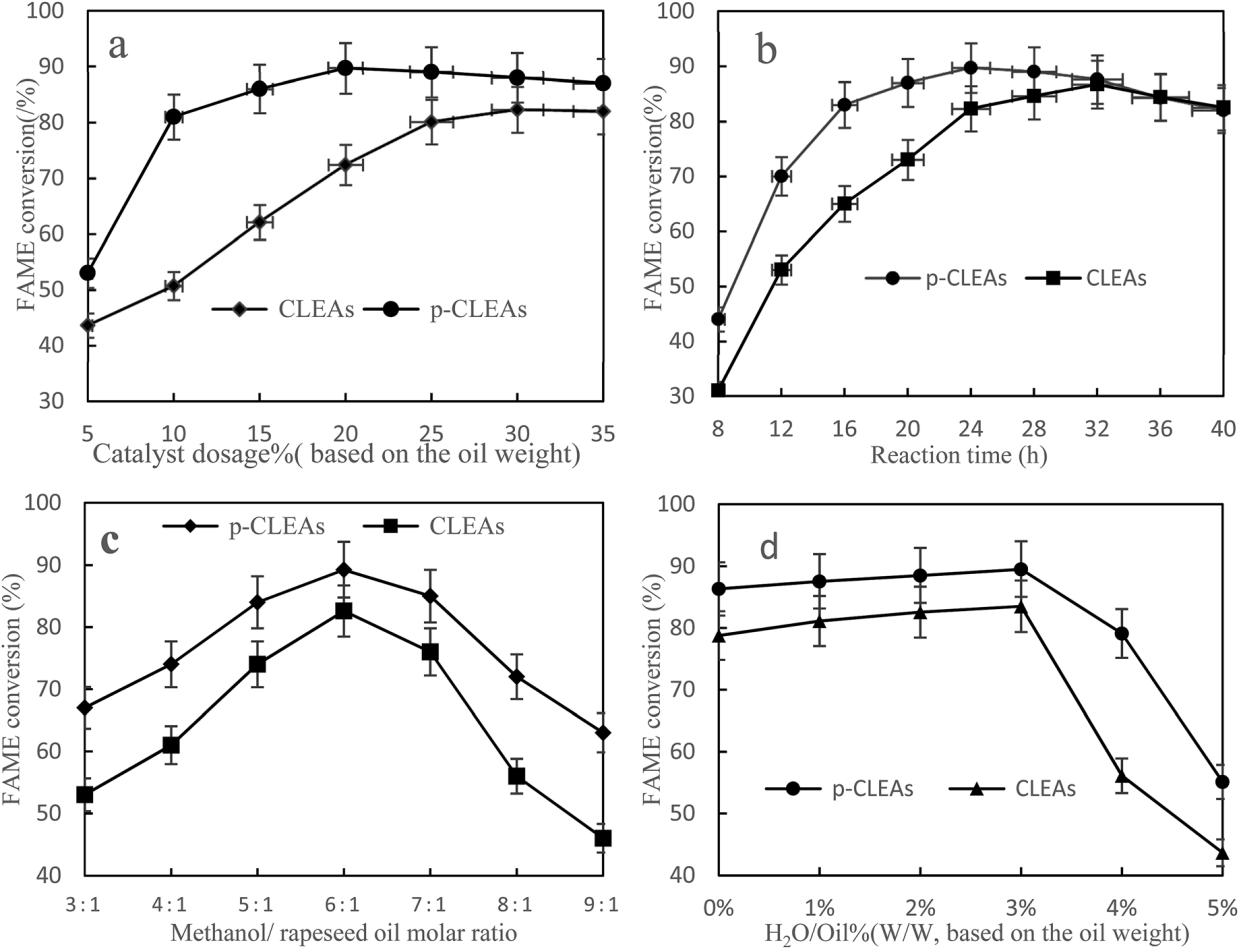
Effects of (a) methanol/oil molar ratio, (b) catalyst dosage, (c) reaction time, and (d) water content on FAME conversion.

Fig. 9a shows the FAME conversion when different p-CLEAs lipase catalyst dosages were used. As indicated in this figure, the FAME conversion increases with the increase in lipase catalyst dosage and then, the conversion reaches a stable equilibrium for both the p-CLEAs lipase and the conventional CLEAs lipase. Under the same conditions, the p-CLEAs lipase performed better than the conventional CLEAs lipase. The highest FAME conversion (89.7%) was obtained when the p-CLEAs lipase catalyst dosage was about 20%. By contrast, the highest FAME conversion (82.3%) was obtained when the conventional CLEAs lipase catalyst dosage was about 30%. About 7.4% increase in the conversion of p-CLEAs was obtained compared to that for the conventional CLEAs. Besides, the p-CLEAs lipase catalyst dosages were reduced by 10% than that of conventional CLEAs at the highest methyl ester formation. On investigating the reason, this is presumed to be because of the porous structure of p-CLEAs that dramatically improves the conversion efficiency and reduces the lipase catalyst dosage. This might be because the conventional CLEAs lipase particles have a large size, small specific surface area, smooth outer surface, and compact interior surface, leading to severe steric hindrance, which limits the mass transfer of the substrate within the CLEAs lipase. As a result, the substrate could only react with the outer surface lipase of CLEAs, resulting in low catalytic efficiencies. By contrast, the p-CLEAs lipase was synthesized *via* combined CaCO_3_ templating and exhibited a controllable particle size and pore structure. This porous structure increased the catalytic specific surface of the CLEAs lipase particles, resulting in high catalytic efficiency. Moreover, the porous structure also decreased the steric hindrance to the substrate. Thus, the substrate could easily approach the active sites in inner CLEAs lipase with less mass transfer limitations. Thus, the p-CLEAs lipase showed higher catalytic activities.

Fig. 9b shows that with the increase in reaction time, the conversion rate of biodiesel shows an obvious increasing trend. Compared with the result for conventional CLEAs, it takes less time for the reaction to reach basic equilibrium under p-CLEAs conditions, and the biodiesel conversion rate under p-CLEAs conditions is much higher than that for conventional CLEAs when they are used as the catalyst. As can be seen from Fig. 8b, when the p-CLEAs lipase is used as the catalyst, the FAME conversion reaches over 70% within 12 h, and the highest conversion (89.7%) is achieved at 24 h. In contrast, in the case of conventional CLEAs, the highest conversion (84.4%) was achieved at 36 h. After a 40 h reaction, the biodiesel conversion rates under different catalyst conditions (conventional CLEAs or p-CLEAs) were not significantly different, and the final conversions of FAME were almost the same. This result indicated that the p-CLEAs lipase as a catalyst can only accelerate the reaction rate, but cannot improve the final biodiesel conversion. This could be because compared to p-CLEAs, due to internal mass transfer constraints, it was difficult for the substrates to reach the CLEAs lipase active site, which affected the catalytic property of lipase and thus affected its efficiency. However, when the time was long enough, the same biodiesel conversion rate was eventually obtained for both p-CLEAs lipase and CLEAs lipase as the catalysts.

It is well-known that 1 mol of rapeseed oil can be reacted with 3 mol of methanol to produce 1 mol of glycerol and 3 mol of biodiesel through transesterification. At the same time, since transesterification is a reversible reaction, in order for the reaction to proceed towards the product side, more amount of the solvent must be utilized; excess methanol can enhance the conversion to methyl esters by shifting the equilibrium favorably towards the forward direction. Thus, the effect of the methanol/oil molar ratio on FAME conversion is shown in Fig. 9c; the FAME conversion increased with oil/methanol ratio from 1 : 3 to 1 : 6 and the highest methyl ester yield (89%) could be obtained at the oil/methanol molar ratio of 1 : 6. However, further increase in the methanol concentration resulted in a decrease in the formation of esters due to enzyme inactivation. In addition, the concentration of rapeseed oil was decreased due to the dilution effect of methanol. Thus, the collision frequency of oil and lipase was reduced, and the reaction rate also decreased. Therefore, the optimal reaction molar ratio of methanol and rapeseed oil for the production of biodiesel was 6 : 1.

Water acts as a ‘lubricant’ of polypeptide chains, thus conferring the enzyme with necessary mobility to exert its catalytic action. Therefore, the effect of the initial water concentration on the transesterification reaction was investigated by the addition of water ranging from 0 to 5% (v/v) of the total amount of the reaction mixture. The results in Fig. 9d show that lipase also has a good catalytic effect without water. Under the conditions of a low water amount, water had little influence on the preparation of biodiesel catalyzed by lipase, and the conversion rate of biodiesel increased slightly with the increase in lipase, which indicated that lipase has strong adaptability. However, when the amount of water exceeded 3%, the conversion rate started to decline. This could be because the addition of water increased the amount of water available for the oil to form an oil–water interfacial area; thus, the catalytic activity of the lipases increased. However, excess water may also stimulate the competing hydrolysis reaction; the equilibrium then shifted to the production of free fatty acids. Therefore, 3% amount of water was determined to be the optimal concentration for maximizing the efficiency.

#### 3.5.2 Repeated use of p-CLEAs

**Fig. 10:**
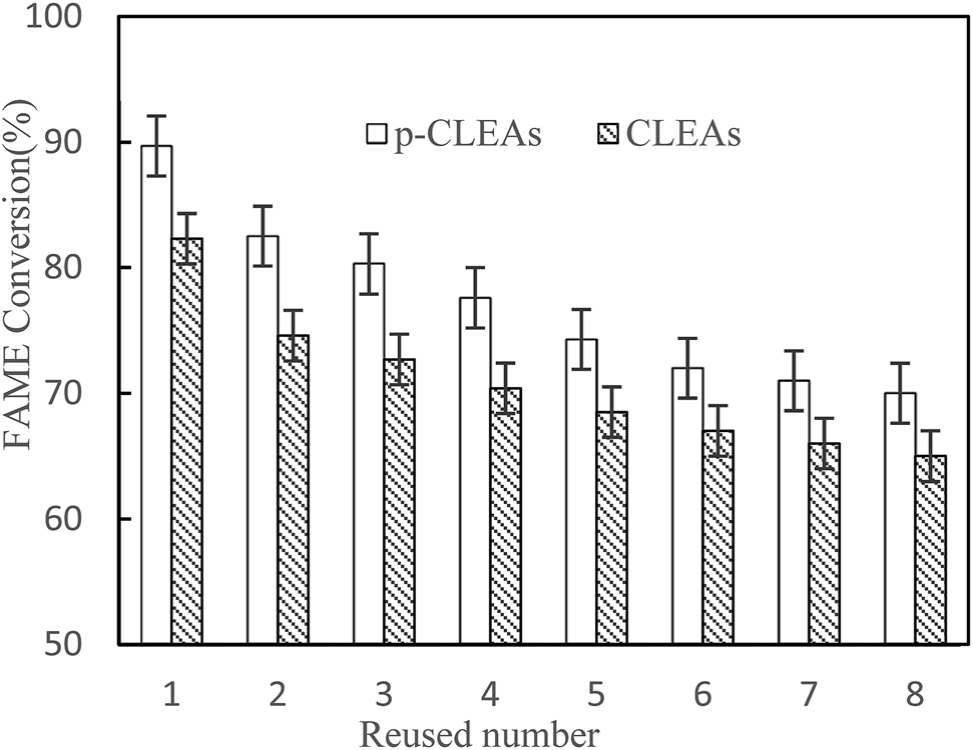
The reusability of the conventional CLEAs and p-CLEAs lipases.

**Fig. 11:**
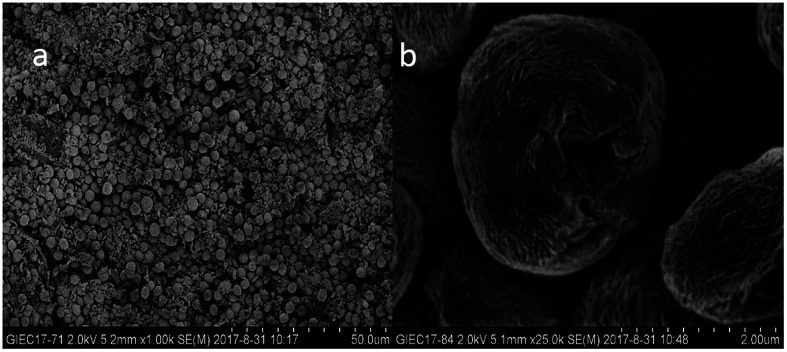
SEM analyses. (a) Some particles were broken; (b) some particles' structure collapsed.

The main advantage of immobilized enzymes is that expensive enzymes can be reused. Thus, the reusability of the p-CLEAs lipase biocatalyst is worth evaluating. In this study, the changes in the recyclability and the stability were studied when p-CLEAs lipase was reused. The changes in the lipase activity are shown in Fig. 10. Both the conventional CLEAs and p-CLEAs lipases exhibited slightly decreased activity in the transesterification reaction upon repeated use after 8 cycles although to different degrees. The p-CLEAs lipase exhibited more superior reusability and stability than the conventional CLEAs. The p-CLEAs lipase after repeated use was used for the transesterification reaction again, and the FAME conversion rate remained above 70% even after 8 times of recycling. In addition, the loss of p-CLEAs lipase activity may be explained in the following three ways: (i) during the reaction procedures, conformational changes occur in lipase. To verify this phenomenon, in this research work, the p-CLEAs lipase was repeatedly used for 8 times and analyzed by SEM. Fig. 11 clearly indicates that with the repeated use of the p-CLEAs lipase, some p-CLEAs lipase particles are broken and although some particles are not broken, the pore structure collapses and the original morphology is no longer maintained. The fragmentation and collapse of the p-CLEAs lipase particles prevented the substrate from entering the pores of the p-CLEAs lipase, thus reducing the lipase catalytic reaction performance. Therefore, in the future work, it is necessary to conduct more in-depth research on improving the structural stability of the p-CLEAs lipase particles. (ii) The toxicity of methanol to p-CLEAs lipase. (iii) Glycerol is adsorbed on the surface of the p-CLEAs lipase, thereby limiting the substrate's contact with the active site of the lipase molecule.^3^

After each batch reaction, the immobilized lipase was recovered by centrifugation and then, a batch of new rapeseed oil and methanol was added to the reactor containing the recovered used lipase. Moreover, fresh lipase was not added to compensate for the loss of lipase in previous rounds. The FAME conversion of each subsequent reaction cycle was analyzed. Reaction conditions: rapeseed oil (10 g), oil to methanol molar ratio 1 : 6, lipase catalyst dosage 20%, water content 3%, temperature 45 °C, and 24 h reaction time.

## 4. Conclusions

The CLEAs technology has been proven to be widely applicable for the immobilization of lipases. However, more research is needed to improve the diffusive resistance of CLEAs and their performance in industrial processes. In this study, p-CLEAs lipase was obtained by co-precipitation with CaCO_3_ as templates and then by DTT induced disulfide bonds in molecule of lipase to form intermolecular disulfide bonds, and finally formed spherical lipase particles. Finally, the CaCO_3_ templates were removed by EDTA to form porous CLEAs. The results of morphological characterization showed that compared with conventional CLEAs, p-CLEAs presented a regular shape, small particles, large pore sizes, low steric hindrance, and large specific surface. Therefore, the mass transfer of the substrates in p-CLEAs was significantly better than that in conventional CLEAs. Additionally, the retention of catalytic conformation as well as structural flexibility by p-CLEAs improved stability in thermal and long-term storage tests, and higher catalytic efficiency was obtained. The p-CLEAs lipase has the potential to be practical and efficient in biodiesel catalysis. Using the p-CLEAs lipase as the catalyst with a methanol-to-oil molar ratio of 6 : 1, a catalyst dose of 20%, and 3% water content at 45 °C for 24 h, the maximum FAME conversion was 89.7%, which remained at about 70% after reusing the catalyst 8 times. However, in the process of the catalytic preparation of biodiesel using p-CLEAs lipase particles as the catalyst, the particles were prone to breakage and collapse, which affected their practical applications. Therefore, in the future work, it is necessary to further study the causes of catalyst loss and improvement measures.

## Conflicts of interest

The authors declared that they have no conflicts of interest to this work. We declare that we do not have any commercial or associative interest that represents a conflict of interest in connection with the work submitted.

## Supplementary Material
